# Development of a prognostic model based on anoikis-related genes for predicting clinical prognosis and immunotherapy of hepatocellular carcinoma

**DOI:** 10.18632/aging.205073

**Published:** 2023-10-02

**Authors:** Mu Pang, Xizhe Sun, Ting He, Huichao Liang, Hao Yang, Jun Chen

**Affiliations:** 1 The Fourth Clinical Medical College of Guangzhou University of Chinese Medicine, Shenzhen Traditional Chinese Medicine Hospital, Shenzhen, Guangdong 518000, China; 2 Research Center for Drug Safety Evaluation of Hainan, Hainan Medical University, Haikou, Hainan 571199, China

**Keywords:** anoikis-related genes, clinical prognosis, immune infiltration, drug sensitivity, hepatocellular carcinoma

## Abstract

Hepatocellular Carcinoma (HCC) is the predominant cause of cancer-related mortality worldwide. The majority of HCC patients are diagnosed at advanced stages of the disease, with a high likelihood of metastasis and unfavorable prognosis. Anoikis resistance is a crucial factor contributing to tumor invasion and metastasis, although its specific role in HCC remains unclear. Based on the results of univariate Cox regression and least absolute shrink-age and selection operator (LASSO) analysis, a subset of anoikis-related genes (ARGs) significantly associated with overall survival (OS) was identified. A multivariate Cox regression analysis subsequently identified PDK4, STK11, and TFDP1 as three prognostic ARGs, which were then used to establish a prognostic risk model. Differences in OS caused by risk stratification in HCC patients were demonstrated. The nomogram analysis indicated that the ARGs prognostic signature served as an independent prognostic predictor. *In vitro* experiments further confirmed the abnormal expression of selected ARGs in HCC. The association between risk scores and OS was further examined through Kaplan-Meier analysis, CIBERSORT analysis, and single-sample gene set enrichment analysis (ssGSEA). This study is a pioneering effort to integrate multiple ARGs and establish a risk-predictive model, providing a unique perspective for the development of personalized and precise therapeutic strategies for HCC.

## INTRODUCTION

Hepatocellular Carcinoma (HCC) is considered as one of the five leading causes of cancer-related death worldwide [1]. In 2018, China recorded over 466,000 new cases of HCC, which resulted in approximately 422,000 HCC-related deaths [1]. Despite the availability of curative treatment options such as surgical resection, liver transplantation, or radiofrequency ablation for early-stage HCC, the majority of patients are diagnosed with advanced-stage disease, often presenting with distant metastasis or unresectable disease [2]. Therefore, there is a critical need to identify more effective specific biomarkers for HCC.

Anoikis, a special type of apoptosis caused by the loss of proper adhesion of cells, plays a crucial role in maintaining normal tissue integrity and participating in tumor invasion and metastasis. The cause of death of most cancer patients is not local tumor cell proliferation, but tumor metastasis. Cancer cells metastasize to the distance after continuous mutual separation or ECM separation and reattach and proliferate [3]. This process usually results in resistance to anoikis [4–7]. To promote their aggressiveness and metastasis, cancer cells employ a variety of mechanisms to produce anoikis resistance, including cell acidosis, reactive oxygen species (ROS) production, epithelial-mesenchymal transition (EMT), and changes in calcium ion transport pathways [8–11]. Multiple signaling pathways that contribute to cancer development are involved in the regulation of anoikis resistance level [12].

The role of anoikis in HCC has received increasing attention. Induction of anoikis prevents malignant transformation of liver cells by modulating the mTOR/S6K1 signaling axis [13]. In addition, as a critical player in the regulation of HCC carcinogenesis and drug resistance [14], EGFR pathway influences HCC distant metastasis by regulating the anoikis process [15]. Multiple targets play an antitumor role in HCC by weakening anoikis resistance [16–18]. Recently, a study revealed that histidine-rich calcium-binding protein (HRC) increases anoikis resistance and promotes HCC metastasis through the protein kinase RNA-like ER kinase (PERK)-eIF2a-ATF4-CHOP signaling pathway [19]. In another study, inducing autophagy in the acidic environment of HCC enhanced the ability of HCC cells to resist anoikis, and autophagy inhibitors reversed this effect [20]. Despite the evidence that anoikis resistance contributes to the development of HCC and that ARGs play a central role in the progression and metastasis of various tumors [21–24], no reports exist on predicting the prognosis of HCC based on an ARGs risk model.

The liver is rich in innate and adaptive immune cells, which result in an intrinsic immune tolerance [25]. Immune tolerance prevents the liver from overreacting to harmful stimuli. On the other hand, it impedes immune surveillance and promotes tumorigenesis and progression [26]. During the development of HCC, chronic inflammation caused by immune cell infiltration produces changes in the immune microenvironment and induces DNA damage and genetic alterations, thus facilitating the development and progression of tumors [27]. Chronic inflammation is involved in up to 80% of HCC cases [27]. In addition, immune cells in the HCC tumor immune microenvironment provide new targets for the next generation of immunotherapy. Immune checkpoint therapy targeting PD-1/PD-L1 in HCC patients has achieved considerable success (28434648). However, ICI efficacy in HCC patients still needs to be improved, and better understanding of HCC immune microenvironment is needed.

This study systematically investigated the correlation between ARGs and the clinicopathological characteristics of HCC patients based on data from the Cancer Genome Atlas (TCGA) and GEO databases. A novel risk model was established, based on three prognostic ARGs, and its ability to predict outcomes in HCC was further evaluated. Additionally, this study provided comprehensive insight into immune infiltration and underlying signaling pathways in HCC patients with varying risk scores. The purpose of this study is to offer new insights into potential treatment strategies and related metastasis mechanisms for HCC.

## MATERIALS AND METHODS

### Data collection from the TCGA and GEO database

For this study, the transcriptome matrix (TPM), which included survival time, survival status, age, gender, stage, T stage, and N stage, was retrieved from the TCGA database (https://portal.gdc.cancer.gov/). A total of 365 HCC samples were included in the analysis [28]. Perl scripts were utilized to merge the gene expression data of each sample. In addition, 115 HCC samples from the GEO database (GSE76427) were obtained and subjected to further analysis (TPM). To ensure data normalization and remove any batch effects, the R package “sva” was employed [29]. The mutation file (MAF) and copy number variate (CNV) data of HCC were collected from the TCGA database.

### Construction and validation of anoikis-related genes risk model

A total of 33 ARGs were obtained from the Molecular Signatures Database (https://www.gsea-msigdb.org/gsea/) [30] (Supplementary Table 1). Univariate Cox regression analysis and the LASSO algorithm were used to identify the ARGs that could predict the prognosis of HCC. Subsequently, multivariate Cox regression analysis was performed to select the final set of prognostic ARGs. Based on these selected ARGs, a risk model was established to predict the prognosis of HCC. The risk score for each sample was calculated using the following formula: risk score = (−0.185 × PDK4) + (−0.540 × STK11) + (0.412 × TFDP1). The HCC samples were then divided into high- and low-risk groups using the median value of the risk score as the cutoff. The training cohort comprised 365 HCC samples from the TCGA database, while the validation cohort comprised 115 HCC samples from the GEO database. In both cohorts, the risk score for each sample was calculated, and the samples were subsequently divided into low- and high-risk groups based on the median risk score.

### Independent prognosis analysis and consensus clustering analysis

In this study, the “ConsensusClusterPlus” R package was utilized to conduct a consensus clustering analysis based on partitioning around medoids. The analysis was carried out with “euclidean” distances and 1000 verifications, with K values ranging from 2–9. Subsequently, patients with HCC were categorized into different subgroups based on the optimal classification obtained. To determine the independence of the risk model, univariate and multivariate Cox regression analyses were performed using the “survival” R package. Moreover, using the clinicopathological characteristics and risk scores, a nomogram model was developed with the “rms” R package, which enabled the calculation and evaluation of the 1-, 3-, and 5-year survival probabilities for patients with HCC. The diagnostic accuracy of the risk score was evaluated using the “pROC” R package. Additionally, a time-dependent receiver operating characteristic (ROC) analysis was conducted to assess the prognostic capability of the risk model.

### Real-time quantitative RT-PCR (qRT-PCR) and western blot analysis

Total RNA was extracted from the L02 and Huh7 cell lines using Trizol reagent (Cat# 15596018, Thermo Fisher) and subjected to cDNA synthesis using an RT reagent kit with gDNA Eraser (Perfect Real Time) for real-time quantitative qRT-PCR (Cat# RR047A, Takara). The resulting mRNA expression was quantified using SYBR Premix Ex Taq II (TliR-NaseH Plus) (Cat# RR820B, Takara), with gene-specific primer pairs listed in Supplementary Table 2. To extract protein, the L02 and Huh7 cells were lysed with RIPA lysis solution, and the protein concentration was subsequently measured using the BCA method. The protein was then subjected to SDS-PAGE electrophoresis for membrane transfer, electrotransfer, and closure. PDK4 (ab110336, 1:1000), STK11 (TA802377, 1:1000), and TFDP1 (PA5-86135, 1:1000) antibodies were added to the membrane respectively and incubated overnight at 4°C. After washing with TBST buffer solution three times, the transferred membrane was incubated with a secondary antibody (1:20000) at room temperature for one hour and washed with TBST three times. Finally, the protein bands were visualized using an Odyssey Clx (Li-Cor, USA), and the blots were imaged and quantified using ImageJ software, with β-actin used as a loading control.

### Immune infiltration landscape, drug sensitivity and GSEA analysis

In order to evaluate the proportion of immune cells, we employed the single-sample gene set enrichment analysis (ssGSEA) algorithm with the “GSVA” R package. The response to anti-PD1/CTLA4 therapy in patients with HCC was assessed using the Cancer Immunome Atlas (TCIA) database (https://tcia.at/home). To determine drug sensitivity, we utilized the Genomics of Drug Sensitivity in Cancer (GDSC) database and calculated the IC50 values with the “pRRophetic” R package. To investigate the correlation between IC50 values and the risk score, we conducted a Spearman-ranked correlation analysis and presented the results in a heatmap with the “ggplot2” R package. Differential expression genes (DEGs) were identified between the low- and high-risk groups (|Fold Change| ≥ 2 and *P* < 0.05). We conducted a gene set enrichment analysis (GSEA) on the DEGs to enrich them into KEGG pathways based on the reference gene set “c2.cp.kegg.v7.2.symbols”.

### Statistical analysis

In this study, all statistical analyses were conducted utilizing R software (version 4.1.1) and Perl scripts. The Spearman-ranked correlation analysis was utilized to assess the association between ARGs and immune cells, with statistical significance set at *P* < 0.05. The differential functions between the two groups were analyzed using the Wilcoxon rank-sum test, while ANOVA test was used for multiple groups. A statistical significance level of *P* < 0.05 was employed in all analyses.

### Availability of data and materials

The raw data of this study were derived from the TCGA (https://portal.gdc.cancer.gov/) and GEO data portal (https://www.ncbi.nlm.nih.gov/geo/; accession number: GSE76427, which were available from the corresponding authors upon request.

## RESULTS

### Differential expression and somatic mutational analysis of ARGs in HCC

**Figure 1:**
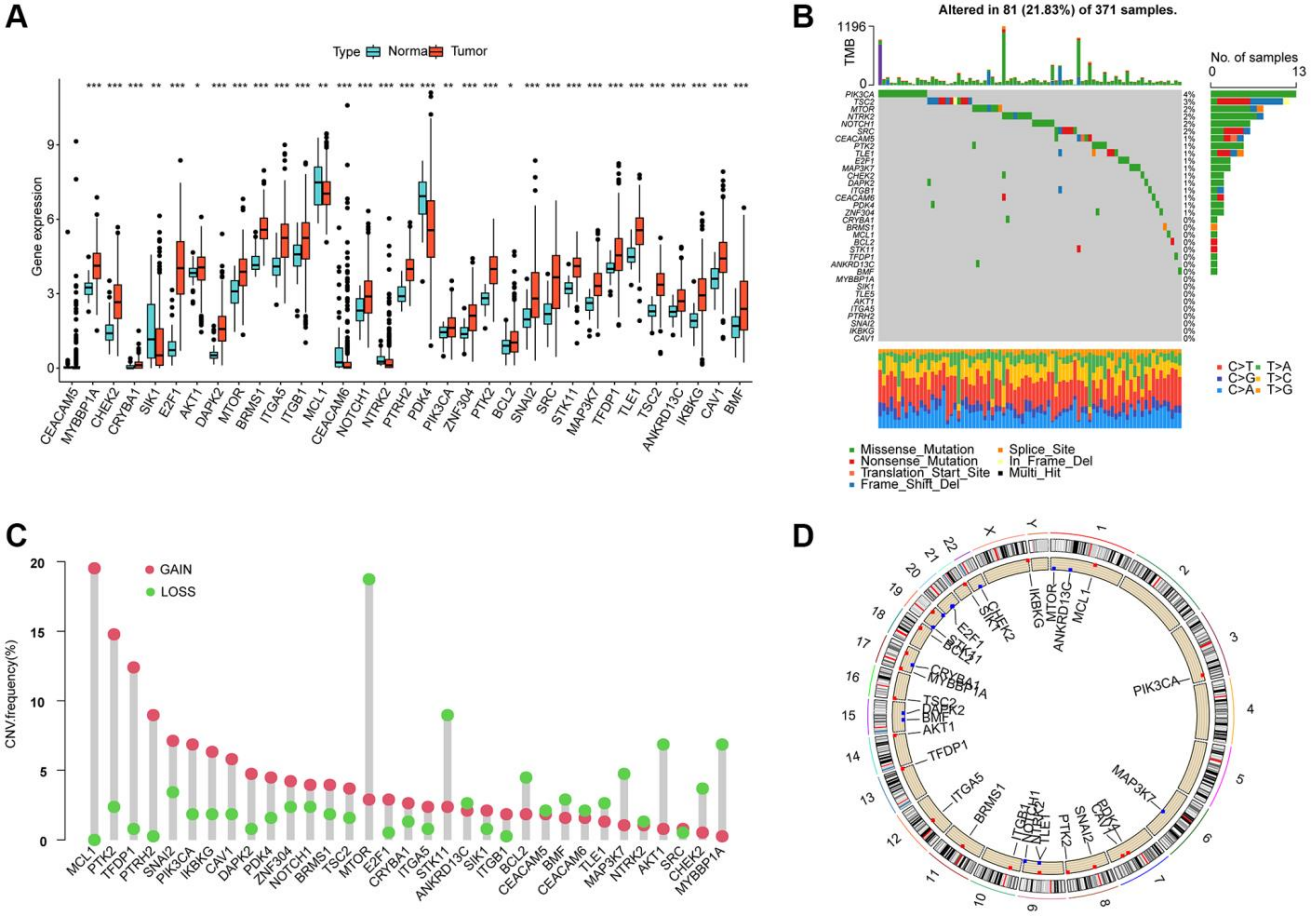
**Differential expression, somatic mutation and CNV analysis of ARGs in HCC.** (**A**) Analysis of differential ARGs expression in normal and HCC tissues. (**B**) Somatic mutation frequency analysis of ARGs in HCC. (**C**) Analysis of copy number variation of ARGs in HCC. (**D**) The circle diagram shows the location of the 33 ARGs on the chromosome.

In this study, a total of 33 ARGs were collected from the MSigDB database, and their potential role in HCC was investigated. To evaluate their expression profiles in normal and HCC tissues, we used “limma” script and conducted differential expression analysis. The results revealed that 32 ARGs were significantly differentially expressed in normal and HCC tissues (Figure 1A, *p* < 0.05). Furthermore, the somatic mutation waterfall plot displayed the mutation frequency of ARGs in HCC, and the analysis indicated that PIK3CA, TSC2, MTOR, SRC, NOTCH1, and NTRK2 had mutation frequencies of 4%, 3%, 2%, 2%, 2%, and 2%, respectively (Figure 1B). Additionally, the copy number analysis of ARGs in HCC showed significant amplification of most ARGs, such as MCL1, PTK2, TFDP1, PTRH2, SNAI2, and PIK3CA, while several genes including MTOR, STK11, BCL2, MAP3K7, AKT1, CHEK2, and MYBBP1A were significantly missing (Figure 1C). Moreover, we also explored the chromosomal location of the 33 ARGs (Figure 1D). The findings suggest substantial differences in ARG expression, mutation, and copy number variation in HCC, thus highlighting their potential role in HCC.

### Molecular subtype profiling of HCC based on prognostic ARGs

**Figure 2:**
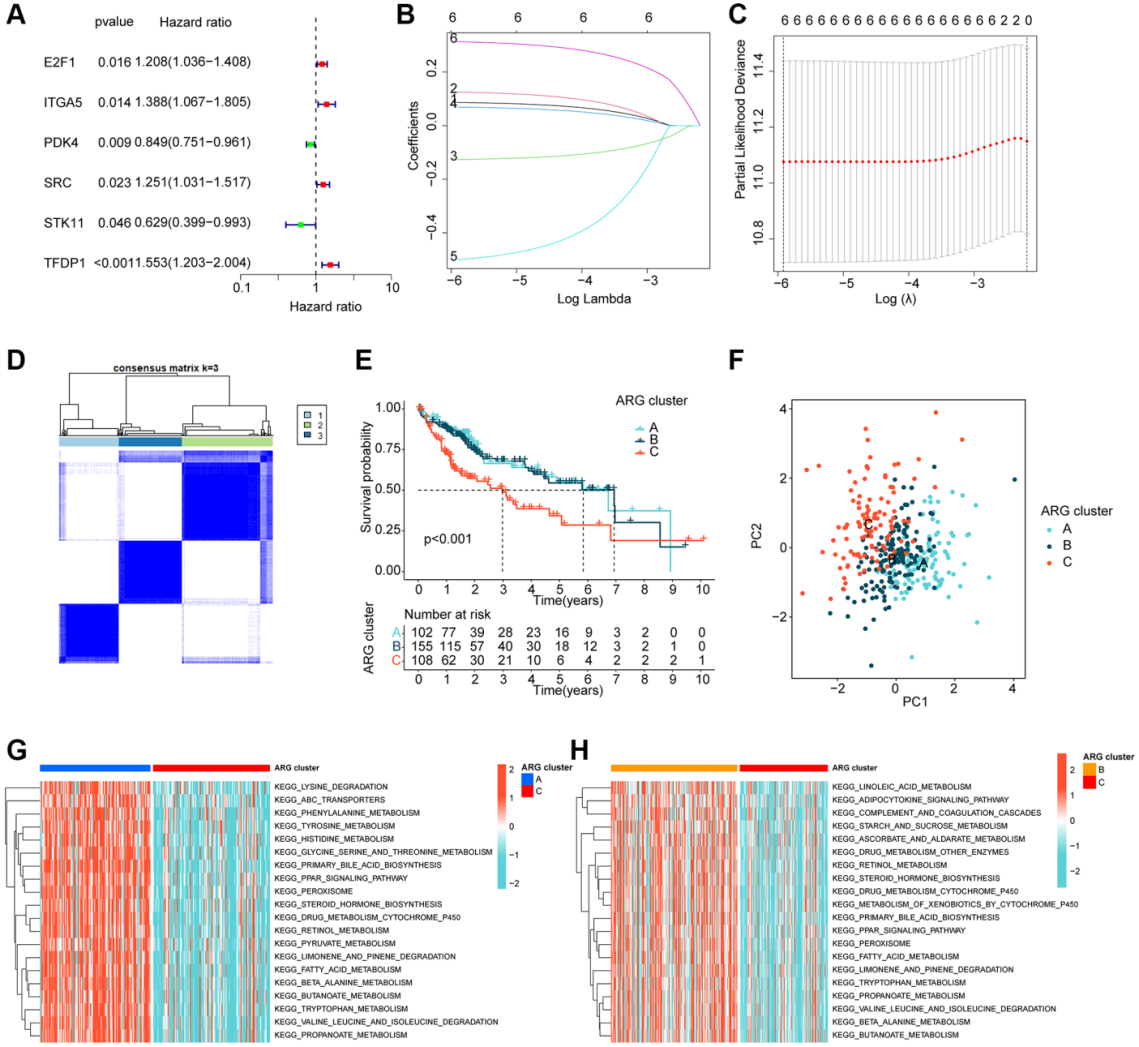
**ARG-based molecular subtyping of HCC.** (**A**–**C**) Identification of prognostic ARGs clinically relevant to HCC based on LASSO-univariate Cox analysis. (**D**) ARG-based analysis of HCC molecular subtypes. (**E**) Clinical survival outcome analysis of HCC samples with different molecular subtypes. (**F**) Unsupervised PCA analysis. (**G**, **H**) GSVA analysis of molecular subtypes of HCC.

Using LASSO-univariate Cox analysis, we identified 6 ARGs that were associated with HCC prognosis, consisting of 4 risk factors (E2F1, ITGA5, SRC, TFDP1) and 2 favorable factors (PDK4, STK11) (Figure 2A–2C). Furthermore, multivariate Cox analysis was carried out to determine 3 independent prognostic factors that were associated with the clinical prognosis of HCC. To explore the potential relationship between ARGs and different molecular subtype characteristics of HCC, we conducted consensus cluster analysis based on the 3 independent prognostic factors of HCC, which demonstrated that HCC samples can be accurately classified into 3 different molecular subtypes when k = 3, with cluster A containing 102 HCC samples, cluster B containing 155 HCC samples, and cluster C containing 108 HCC samples (Figure 2D). It was observed that the clinical survival outcome of HCC in cluster C was significantly worse than that of cluster A and B, while the clinical survival outcome was similar between cluster A and cluster B (Figure 2E, *p* < 0.001). Moreover, unsupervised PCA plots displayed a significant variability among the 3 HCC molecular subgroups (Figure 2F). To further investigate the potential molecular mechanisms between the different ARG-based molecular subtypes, GSVA results illustrated that metabolism-related pathways were considerably downregulated in Cluster C compared to Cluster A and Cluster B, encompassing tyrosine metabolism, pyruvate metabolism, and fatty acid metabolism (Figure 2G, 2H).

### Immune infiltration and immunotherapeutic response analysis of molecular subtypes of HCC

**Figure 3:**
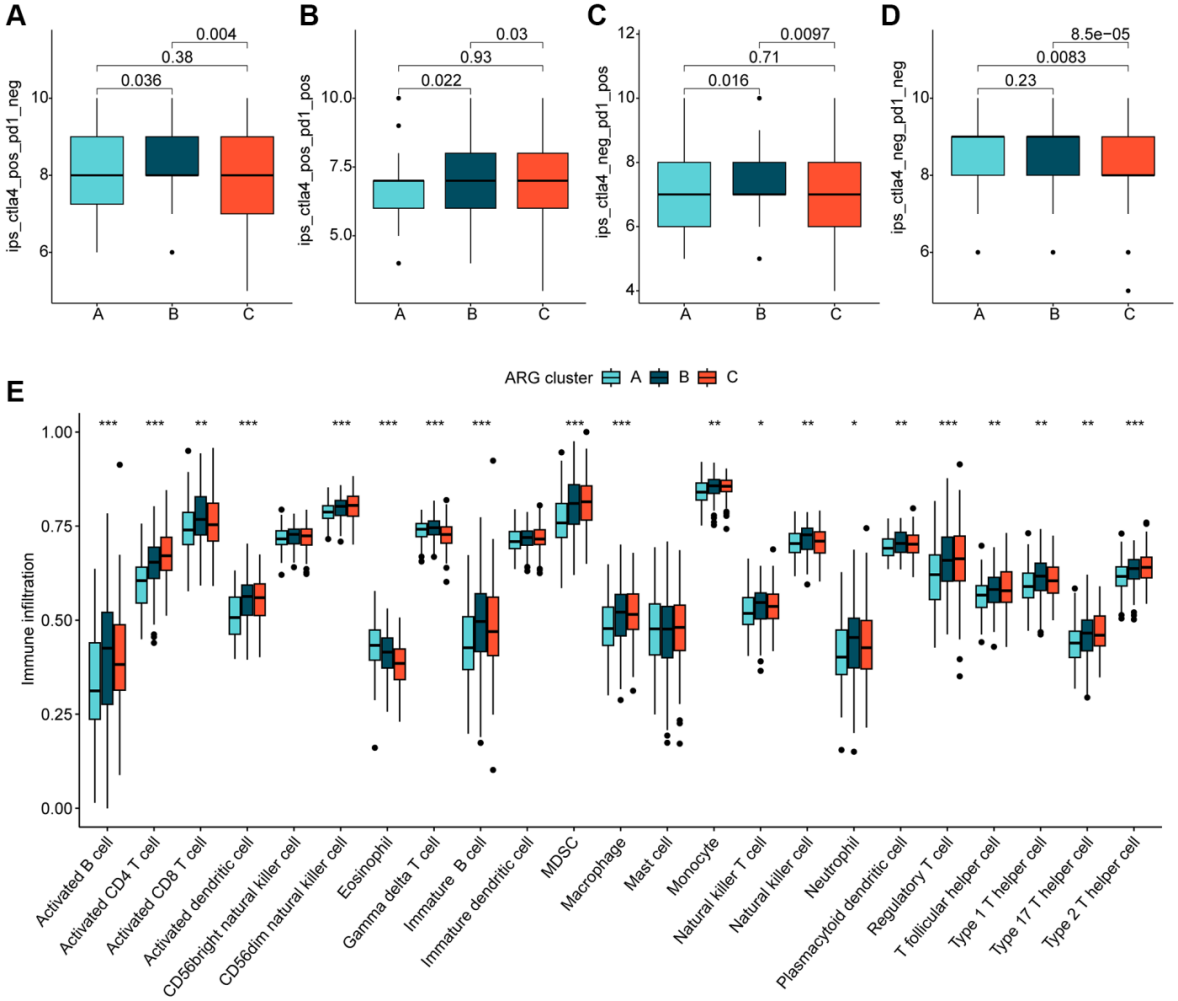
**Immune infiltration of HCC molecular subtypes and immunotherapy assessment analysis.** (**A**–**D**) IPS analysis reveals the therapeutic response of different HCC molecular subtypes to PD-1 and CTLA-4. (**E**) Immune infiltration landscape of HCC molecular subtypes assessed based on the ssGSEA algorithm. Statistical significance: ^*^p < 0.05, ^**^p < 0.01, ^***^p < 0.001.

We further investigated the relationship between the different molecular subtypes of HCC and immune infiltration. Our results from the IPS analysis indicated that HCC samples from cluster B may respond better to immunotherapy with PD-1 and CTLA4 (Figure 3A–3D). Additionally, using the ssGSEA algorithm, we evaluated the immune infiltration landscape of the different HCC molecular subtypes. The findings from the immune infiltration assessment showed that most of the immune cell proportions were significantly higher in cluster B than in clusters A and C. These immune cell types included activated B cells, CD8+ T cells, immature B cells, neutrophils, type 1 T helper cells, and gamma delta T cells (Figure 3E). In summary, our results demonstrate a significantly different immune infiltration landscape between the various HCC molecular subtypes, which is closely associated with immunotherapeutic response.

### Risk model construction for HCC

**Figure 4:**
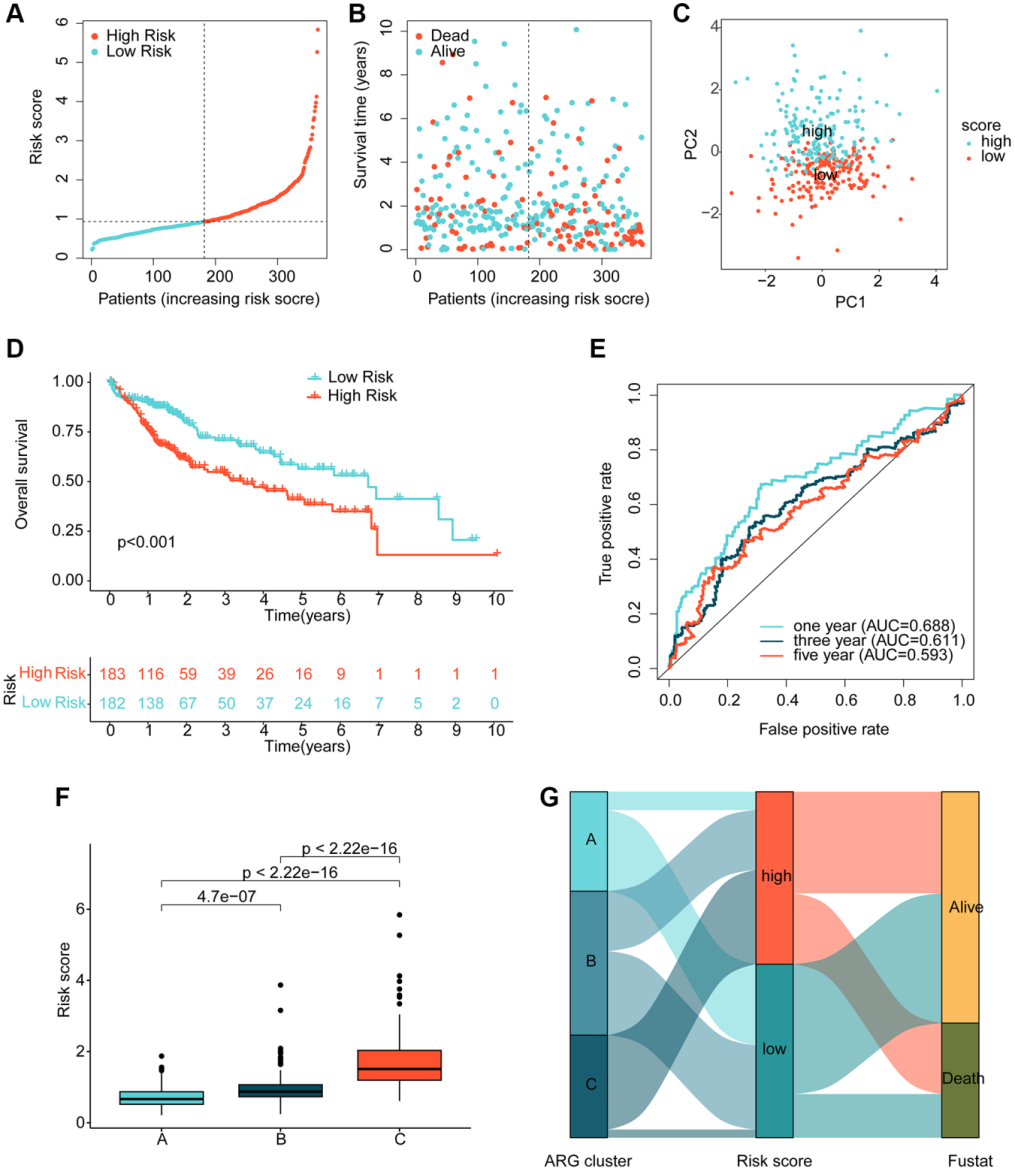
**Risk model development based on prognostic ARGs.** (**A**, **B**) Subgroup analysis of HCC risk based on prognostic ARGs. (**C**) Unsupervised PCA analysis. (**D**) Clinical survival outcome assessment of HCC risk subgroups. (**E**) Time-dependent ROC curve analysis. (**F**) Risk score distribution of HCC molecular subtypes. (**G**) Association analysis of HCC molecular subtypes, risk subgroups and clinical survival outcomes.

Using multivariate Cox analysis, we obtained risk coefficients and expression profiles of 3 independent prognostic factors, which we then used to calculate risk values for each HCC sample in the TCGA. HCC samples were subsequently divided into low- and high-risk subgroups based on their risk values. Scatter plots of the risk model indicated that HCC samples in the high-risk group contained more deaths (Figure 4A, 4B). Notably, unsupervised PCA analysis demonstrated two clear patterns of distribution of HCC risk subgroups (Figure 4C). Clinical survival outcome curves suggested that clinical prognostic outcomes were significantly better for HCC samples in the low-risk subgroup than in the high-risk subgroup (Figure 4D, *p* < 0.001). Time-related ROC curves suggested AUC of 0.688, 0.611, and 0.593 for 1-, 3-, and 5-years, respectively (Figure 4E). We also observed significantly higher risk scores for HCC samples in cluster C, which had the worst clinical prognosis, compared to clusters A and B (Figure 4F). Sankey plots demonstrating the association of HCC molecular subtypes and risk subgroups with clinical prognosis showed that HCC samples in cluster C were more likely to be classified into higher risk subgroups and were associated with poor clinical survival outcomes (Figure 4G).

### Prognostic ARG-based risk model validation

**Figure 5:**
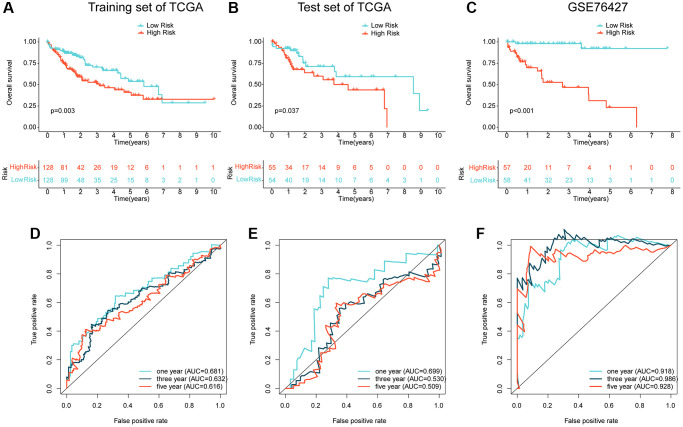
**Independent cohort validation of the risk model developed based on the prognostic ARG.** (**A**, **B**) Clinical prognostic analysis of HCC samples from the training and validation sets in the TCGA cohort. (**C**) Clinical prognostic analysis of HCC samples from the GSE76427 dataset. (**D**–**F**) Time-related ROC curve analysis of the TCGA cohort and the GSE76427 cohort at 1-, 3-, and 5-year.

To validate the independence and reliability of the prognostic ARG-based risk model, we conducted both internal and external validation analyses using the TCGA dataset and GSE76427 dataset, respectively. Using the “caret” script, we randomly partitioned the HCC samples in TCGA into training and validation sets with a 7:3 ratio and calculated risk values for each sample in both sets. Our findings demonstrated that the clinical prognostic outcomes of the HCC samples in the low-risk subgroup were significantly better than those in the high-risk subgroup in both the training and validation sets, as shown in Figures 5A, 5B. Furthermore, in the external validation set, GSE76427, we observed consistent results where HCC samples in the high-risk subgroup exhibited worse clinical prognosis than those in the low-risk subgroup (Figure 5C). Time-dependent ROC curves showed AUC of 0.681, 0.632, and 0.616 for 1-, 3-, and 5-year predictions, respectively, in the training set, AUC of 0.699, 0.530, and 0.509 in the validation set, and AUC of 0.918, 0.986, and 0.928 in GSE76427 (Figure 5D–5F). Our results demonstrate that the ARG-based risk model can accurately and independently predict the clinical prognosis of HCC.

### Independent prognostic assessment of risk scores in an independent cohort

**Figure 6:**
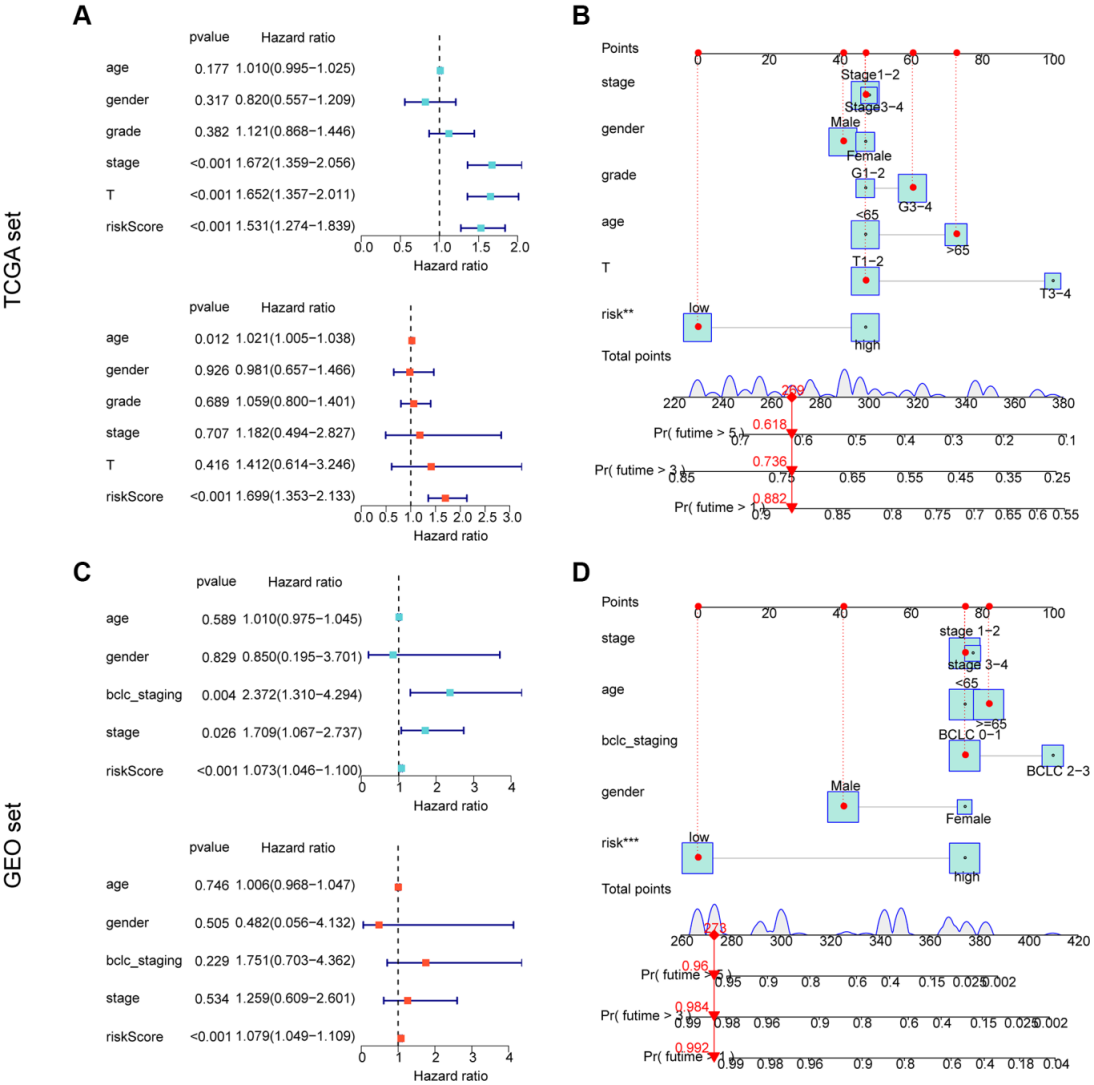
**Independent prognostic assessment and nomogram development of risk models based on ARG constructs in different independent cohorts.** (**A**, **B**) Independent prognostic analysis and nomogram construction in the TCGA cohort. (**C**, **D**) Independent prognostic analysis and nomogram construction in the GEO cohort.

We conducted further analysis to assess the independent prognostic value of the risk model developed based on the prognostic ARGs in combination with other clinicopathological characteristics. Using the TCGA cohort, we performed univariate analysis and found that stage (HR = 1.672 (.359–2.056), T (HR = 1.652 (1.357–2.011), *p* < 0.001), and risk score (HR = 1.531 (1.274–1.839), *p* < 0.001) were significantly associated with poor prognosis of HCC. Multifactorial Cox analysis indicated that the risk score (HR = 1.699 (1.353–2.133)) was an independent prognostic factor for HCC (Figure 6A). We developed a nomogram based on the risk scores and clinicopathological characteristics of the TCGA cohort, which accurately predicted the 1-, 3-, and 5-year survival probabilities of HCC samples (Figure 6B) (Supplementary Figure 1A, 1B). For the GEO cohort, one-way Cox analysis showed that bclc-staging (HR = 2.372 (1.310–4.294), *p* = 0.004), stage (HR = 1.709 (1.067–2.737), *p* = 0.026), and risk score (HR = 1.073 (1.046–1.100), *p* < 0.001) were significantly associated with poor prognosis of HCC. Multivariate Cox analysis indicated that the risk score (HR = 1.079 (1.049–1.109), *p* < 0.001) was an independent prognostic factor for HCC (Figure 6C). We developed a nomogram based on the risk scores and clinicopathological characteristics of the GEO cohort, which accurately predicted the 1-, 3-, and 5-year survival probabilities of HCC samples (Figure 6D) (Supplementary Figure 1C, 1D). In conclusion, our results indicate that the ARG-based risk model is an independent prognostic factor for HCC samples, which is independent of clinicopathological features.

### Mutational burden and GSEA analysis of risk subgroups

**Figure 7:**
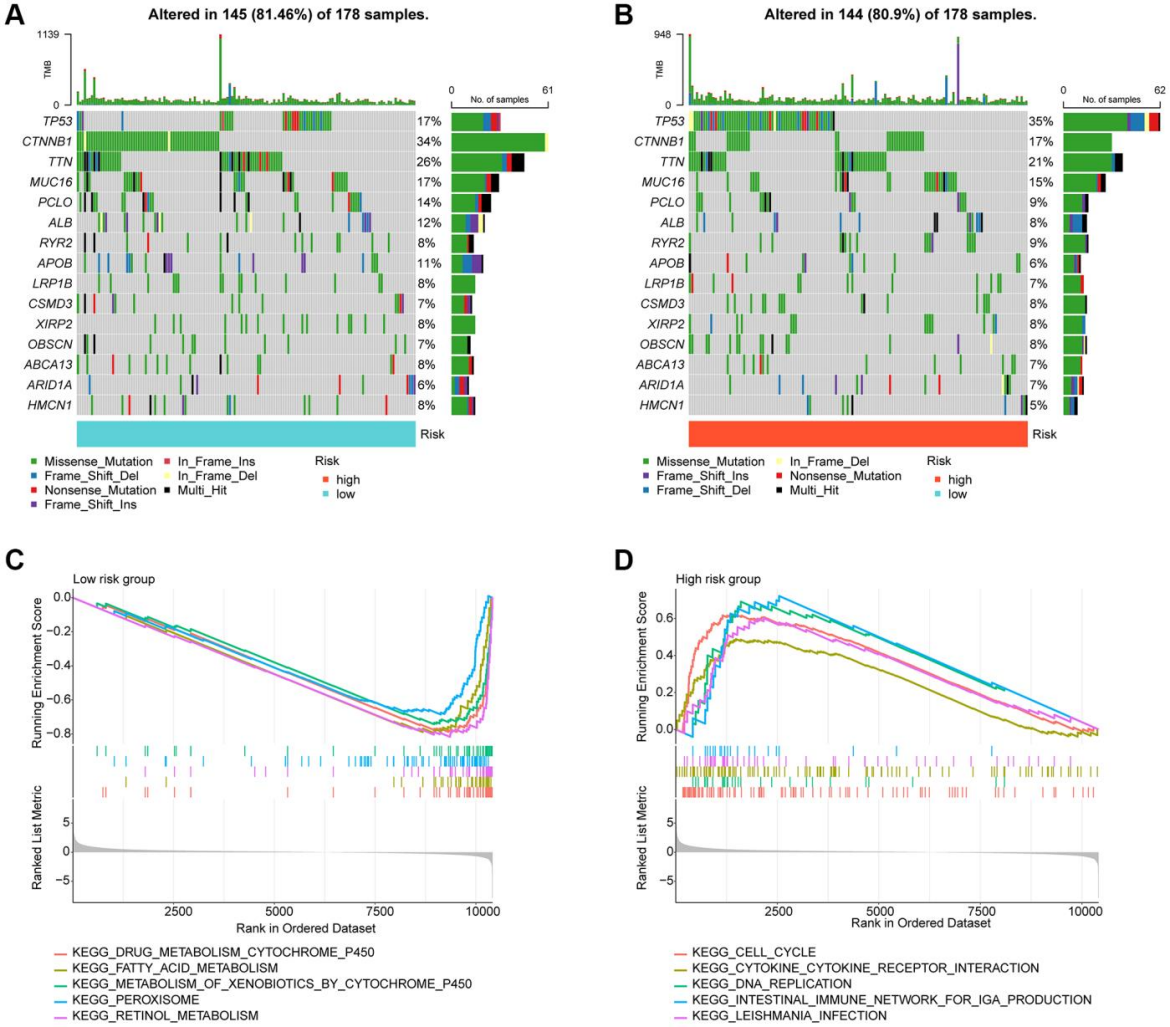
**Tumor mutational burden and GSEA analysis in risk subgroups.** (**A**, **B**) Somatic mutation landscape analysis in the risk subgroup. (**C**, **D**) GSEA analysis of risk subgroups.

In the subsequent study, we delved deeper into the landscape of mutational load in HCC among the risk subgroups. Our findings showed that out of 178 samples in the low-risk subgroup, somatic mutations occurred in 145 (81.46%) samples, while in the high-risk subgroup, significant somatic mutations were present in 144 samples (80.9%). As depicted in Figure 7A, 7B, we observed that the frequency of mutations in TP53 was significantly higher in the high-risk subgroup compared to the low-risk subgroup. Conversely, the frequency of mutations in ALB, PCLO, MUC16, TTN, and CTNNB1 was significantly lower in the high-risk subgroup compared to the low-risk subgroup. To gain a better understanding of the potential regulatory mechanisms in different risk subgroups, we analyzed the KEGG pathway in the high- and low-risk subgroups based on GSEA. Our results suggested a significant enrichment of metabolism-related signaling pathways in the low-risk subgroup, which included drug metabolism cytochrome p450 and fatty acid metabolism. In contrast, the high-risk subgroup showed a significant enrichment of tumor and immune-related signaling pathways such as cell cycle, DNA Replication, and cytokine-cytokine receptor interaction (Figure 7C, 7D).

### Immune infiltration landscape and drug sensitivity analysis in risk subgroups

**Figure 8:**
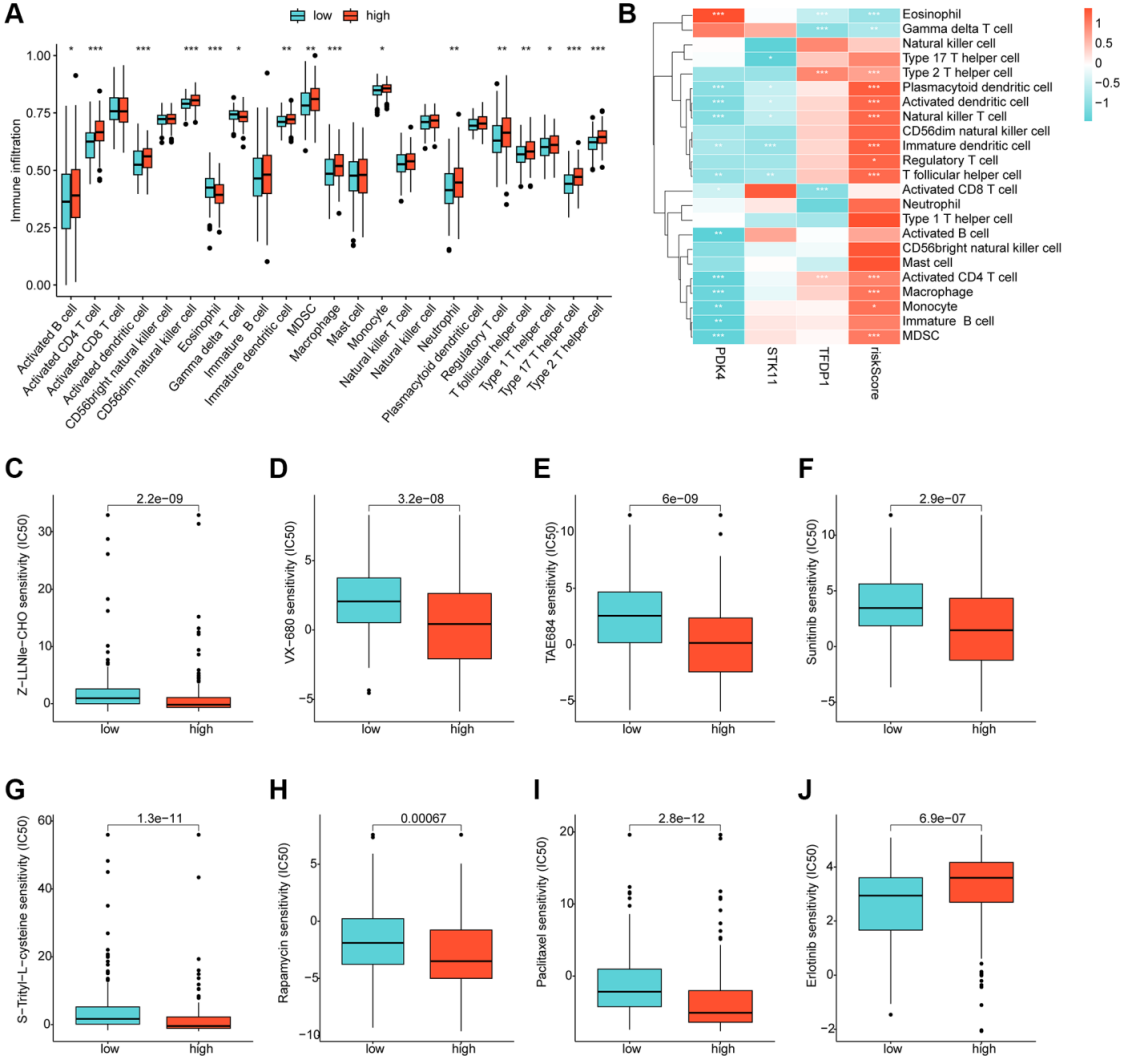
**Immune infiltration landscape and drug sensitivity analysis for risk subgroups.** (**A**) Immune cell proportion of risk subgroups assessed based on the ssGSEA algorithm. (**B**) Correlation analysis of independent prognostic factors and risk scores with immune cells. (**C**–**J**) Predictive analysis of drug sensitivity in risk subgroups.

Using ssGSEA, we conducted a further analysis of the immune infiltration characteristics of HCC in the risk subgroups. Our findings indicated a significantly higher proportion of most immune cells in the high-risk subgroups compared to the low-risk subgroups, signifying a greater immune infiltration status of HCC in the former group (Figure 8A). Correlation analysis showed a significant positive correlation between the risk score and most immune cells, including MDSC, CD4+ T cells, macrophage, monocyte, and immature dendritic cells, while a significant negative correlation was observed with eosinophil and gamma delta T cells. We also observed significant correlations of PDK4, STK11, and TFDP1 with the majority of immunity (Figure 8B). Furthermore, we utilized the GDSC database to predict potential chemotherapeutic agents for HCC samples. Our results demonstrated significantly lower IC50 values for Z-LLNle-CHO, VX-680, TAE684, Sunitinib, S-Trityl-L-cysteine, Rapamycin, and Paclitaxel in the high-risk group compared to the low-risk group, whereas the IC50 for Erlotinib was significantly higher in the high-risk group (Figure 8C–8J).

### qRT-PCR and western blot analysis

**Figure 9:**
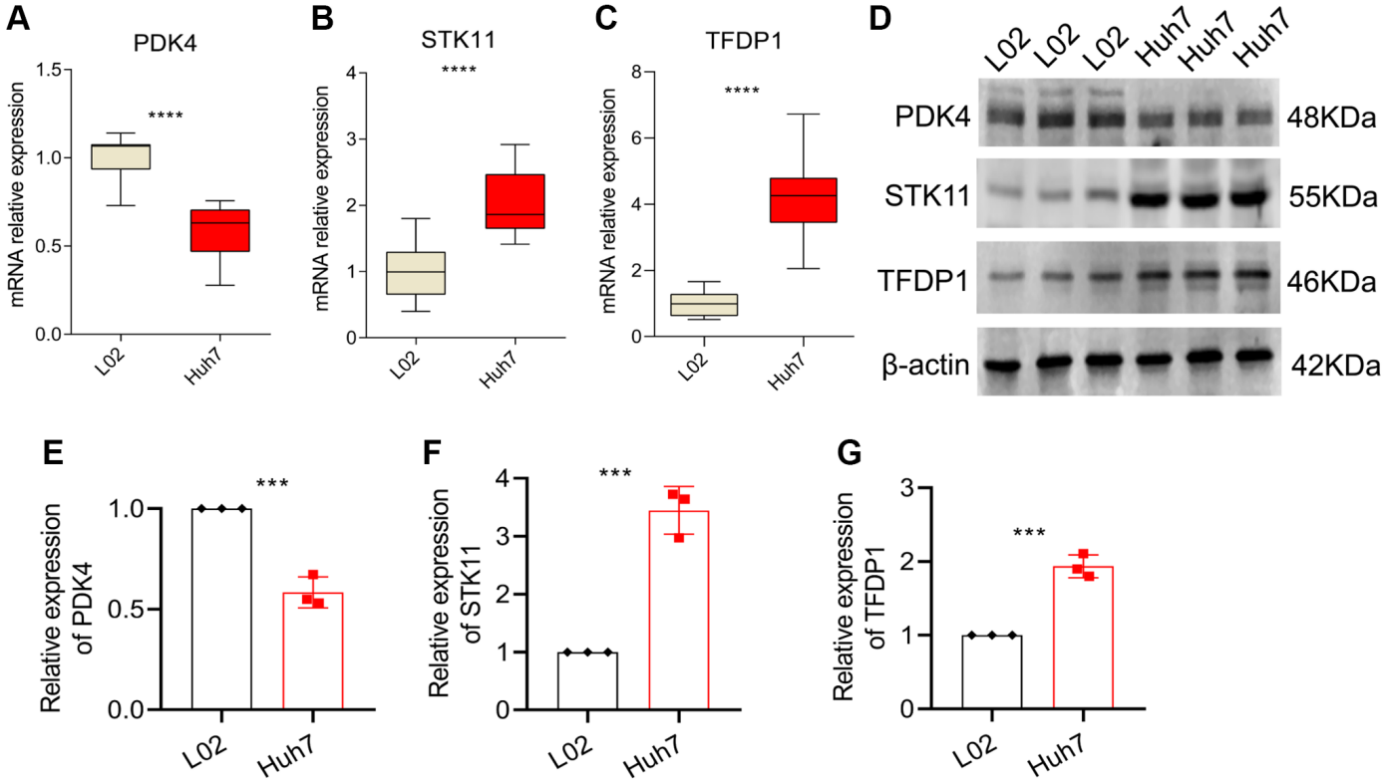
**qRT-PCR and Western blot analysis.** (**A**–**C**) mRNA expression levels of PDK4, STK11 and TFDP1 in L02 and Huh7 cell lines. (**D**–**G**) Western blot and quantitative analysis. Data representation: mean ± SD. Statistical significance: ^*^p < 0.05, ^**^p < 0.01, ^***^p < 0.001, ^****^P < 0.0001.

In order to validate our findings, we conducted further *in vitro* experiments to assess the mRNA and protein expression levels of PDK4, STK11 and TFDP1. Our results showed that compared to the normal cell line L02, the expression of PDK4 was significantly lower, while the expression of STK11 and TFDP1 was significantly higher in the HCC cell line Huh7 (Figure 9A–9C). Western blot analysis also confirmed our findings, as the protein levels of PDK4 were significantly higher in the L02 cell line, while STK11 and TFDP1 were significantly higher in the Huh7 cell line (Figure 9D–9G).

## DISCUSSION

Hepatocellular carcinoma (HCC) accounts for 90% of liver malignancies and is currently the second cause of the cancer-related death worldwide [31]. In this study, 3 ARGs (PDK4, STK11 and TFDP1) which are associated with OS rate for HCC patients were identified, establishing a new risk model in order to evaluate the prognosis of HCC subsequently. The qRT-PCR and western blot results further confirmed the abnormal expression of these genes in HCC patients. The ARGs risk model was validated as an independent prognosis predictor by univariate and multivariate Cox regression analysis. Additionally, the ARGs risk model was also found to be closely related to tumor immune microenvironment and immunotherapy response for HCC, which might provide a new perspective for the future individualized immunotherapy. Drug sensitivity analysis illustrated a different response to targeted drugs by risk stratification.

Our study provides the first evidence of the significance of anoikis in HCC. In fact, there is some evidence suggesting a role for anoikis in liver cancer. The extracellular environment, especially the extracellular matrix (ECM), provides adhesion and connection support between cells, which promotes cell survival and growth [6]. Loss of cell adhesion may lead to programmed cell death, which is called anoikis [32]. Anoikis can limit cancer progression by preventing the cell from disseminating to distant organs. Therefore, different mechanisms were developed from malignant aggressive tumor to counter anoikis which is inclined to obtain the ability to escape from the primary sites to distant organs or lymph nodes [33]. Multiple pathways can lead to the acquisition of anoikis resistance in HCC. Xia et al. found that histidine-rich calcium binding protein (HRC) could enhance the anoikis resistance and promote the HCC metastasis via protein kinase RNA-like ER kinase (PERK)-eIF2a-ATF4-CHOP signaling axis [19]. Mo et al. found that upregulation of IQGAP1 enhanced anoikis resistance, thus promoting the migration and invasion of HCC cells [34]. Based on these evidences, we hypothesized that ARGs were promising biomarkers of HCC.

Cancer cells often exhibit altered glucose metabolism characterized by a preference for aerobic glycolysis or the Warburg effect [35]. PDK4 regulates mitochondrial glycolysis by phosphorylating pyruvate dehydrogenase (PDH) which controls the generation of reducing equivalents driving respiration [36]. Besides, PDK4 also plays an important role in survival, proliferation, invasion and metastasis of tumor cells [37]. Song et al. reported that inhibiting PDK4 could slow the proliferation of liver cancer [38]. In addition, Lu et al. found that when detached from the matrix, untransformed mammary epithelial cells underwent metabolic reprogramming by markedly upregulating PDK4, thereby inhibiting PDH and attenuating the flux of glycolytic carbon into mitochondrial oxidation, and depletion of PDK4 increased mitochondrial respiration and oxidative stress in suspended cells, resulting in heightened anoikis [39]. These results suggested that PDK4 was a potential target for anti-metastasis therapy, consistent with our study.

Serine/threonine kinase 11 (STK11) also referred as Liver kinase B1 (LKB1), encodes a 50 kDa evolutionary conserved serine/threonine kinase [40]. LKB1 can phosphorylate and activate several kinases including AMP-activated protein kinase and shows pleiotropic activity in multiple processes, including the cancer pathology related processes, such as energy metabolism, proliferation and apoptosis [41]. Recently, several studies have reported that LKB1 was upregulated in HCC, which was consistent with this study [42].

The TFDP family includes three members, TFDP1, TFDP2 and TFDP3 [43]. TFDP1 acts as a heterodimerization partner for E2F family members of transcription factors and plays an important role in HCC, mainly through affecting the CDK-RB-E2F cell cycle regulation axis [44]. Moreover, TFDP1 has been identified as a c-Myc-targeted gene, which may promote hepatocyte transformation by changing cell cycle control [45]. Kohichiroh Yasui also found that since it promoted tumor cell growth, elevated TFDP1 expression may significantly affect HCC progression [46].

The liver has a unique immune microenvironment where exists immune cells such as Myeloid-derived suppressor cells (MDSC), dendritic cells (DC), tumor associated macrophages (TAMs), natural killer cells (NK), cytotoxic lymphocytes (CTL), and regulatory T cells (Treg). The non-immune cells in liver include cancer related fibroblasts (CAFs), hepatic stellate cells (HSCs), and liver endothelial cells [47]. Both the immune and non-immune cells participate in immune tolerance and response of HCC, and affect its development and prognosis. According to the immune infiltration analysis results, high-risk group had an immune microenvironment consisting of higher levels of activated CD4+ T cells, DC and macrophages. DC located in tumor microenvironment, are called tumor invasion dendritic cells (TIDC), which can present tumor antigen to initial T cells and induce specific anti-tumor immunity. When the liver is damaged, DC can produce tumor necrosis factor (TNF) to promote T cell proliferation, NK cells activation, leading to inflammation and fibrosis of the liver [48]. The immunosuppressive cells (MDSC) were also significantly elevated in the high-risk group. IL-17 plays an important role in the immune tolerance of MDSC. The loss of IL-17 receptor inhibits the infiltration of MDSC and promotes the infiltration of CD8+ T cells. Macrophages can chemotactic MDSC to tumor and promote their release of IL-17. By contrast, Tregs, which could induce immune escape, had no differences between the low- and high-risk group. Overall, the infiltration characteristics of different subgroups according to the risk score may help HCC patients get personalized immunotherapy.

Risk stratification of hepatocellular carcinoma, including risk stratification algorithms and biomarkers, could help improve the effectiveness of HCC treatment by better identifying at-risk individuals [49]. In addition, the combination of valuable hepatocellular carcinoma biomarkers after screening into an array has positive significance in clinical application [50]. Due to the heterogeneity of tumors and individuals, a large enough biomarker pool is needed. Our study provides new targets for risk stratification and further screening. According to present studies, the results of drug sensitivity analysis showed significant differences in the susceptibility to chemotherapeutic or targeted agents in different risk groups. Compared with targeted therapy, immune checkpoint inhibitor (ICI), the most widely used method for immunotherapy of HCC, had a longer OS as the second-line treatment for advanced HCC [51]. However, the response rate of ICI was only 10%~20% [52]. In this study, ICI analysis suggested that the expression of LAG3, CTLA-4, and PD-L1 in high-risk group were significantly higher than that in low-risk group, which indirectly indicated that the anoikis risk score might have a great influence on predicting the effects of immunotherapy. The combination of targeted therapy and immunotherapy for HCC has made remarkable progress in recent years and has broad prospects [53]. The ARGs risk model may help screen more specific targets for combination therapy to improve the clinical efficacy.

In the present study, we established a novel model based on three prognostic ARGs and confirmed its efficacy in prognosis predicting of HCC patients. By immune infiltration landscape and drug sensitivity analysis, the established ARGs risk model showed its potential value in clinical practice. Our study does have limitations. Although we have verified the abnormal expression of ARGs through qRT-PCR and western blot, further mechanism studies on the effect of ARGs are lacking. Additionally, due to the characteristics of bioinformatics analysis, the correlation analysis conclusions in this study lack further causation analysis. Further mechanism studies in the future will help us to better understand the ARGs role in HCC.

## Supplementary Materials

Supplementary Figure 1

Supplementary Tables
