# Supplementary material for: Development of a prognostic model based on anoikis-related genes for predicting clinical prognosis and immunotherapy of hepatocellular carcinoma
Source: Aging (Albany NY). 2023 Oct 2;15(19):10253–71. doi: 10.18632/aging.205073 (PMC10599733; doi:10.18632/aging.205073)
Supplement: Supplementary Figure 1 [file aging-15-205073-s001.pdf]

SUPPLEMENTARY FIGURE

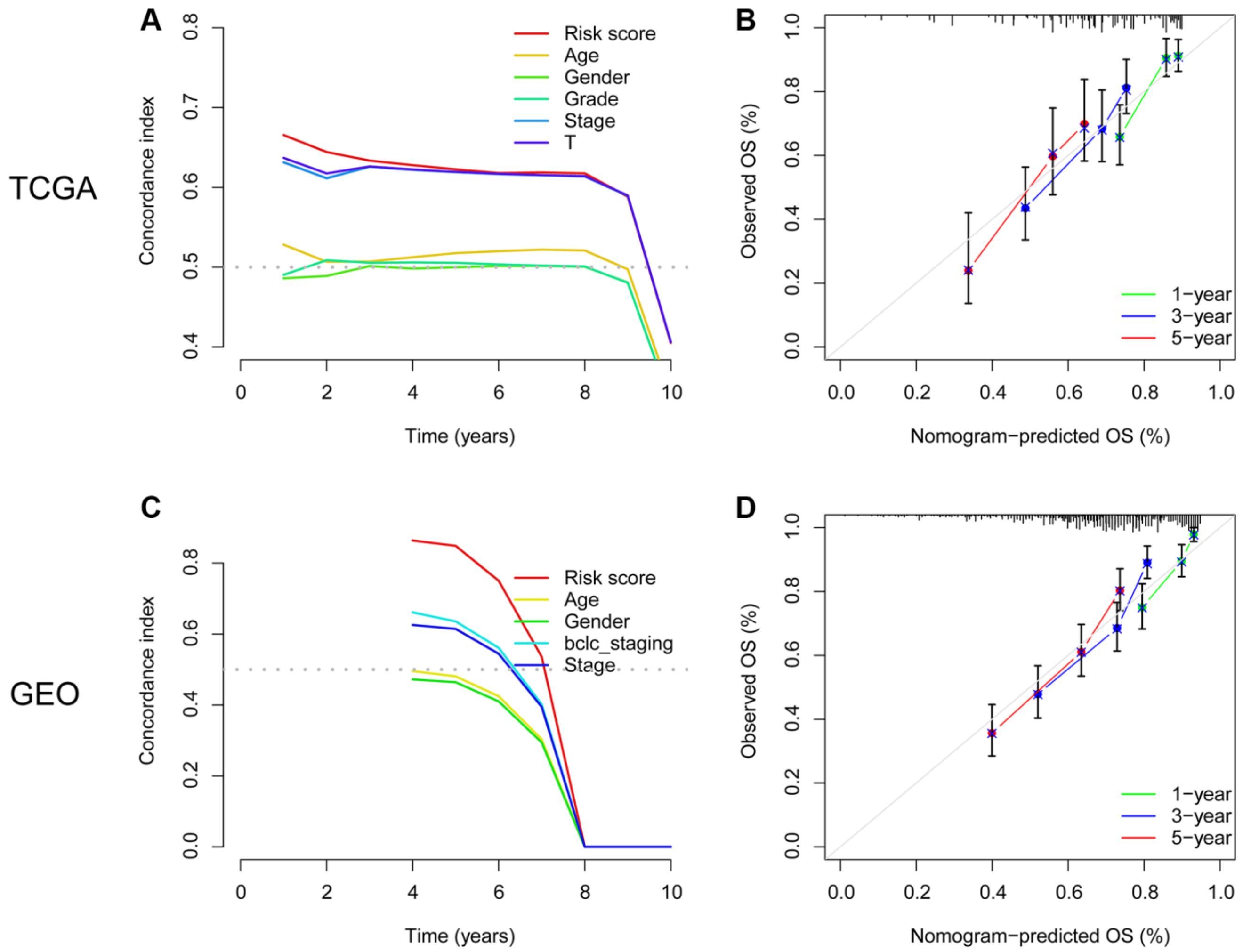

Supplementary Figure 1. The C-index and calibration analysis in TCGA and GEO cohorts. (**A**) Concordance index of TCGA database. (**B**) Calibration curve analysis of TCGA database. (**C**) Concordance index of GEO database. (**D**) Calibration curve analysis of GEO database.
