# Supplementary material for: Development of a prognostic model based on anoikis-related genes for predicting clinical prognosis and immunotherapy of hepatocellular carcinoma
Source: Aging (Albany NY). 2023 Oct 2;15(19):10253–71. doi: 10.18632/aging.205073 (PMC10599733; doi:10.18632/aging.205073)
Supplement: Supplementary Tables [file aging-15-205073-s002.pdf]

SUPPLEMENTARY TABLES

Supplementary Table 1. The gene list of anoikis.

**Anoikis related genes**
CEACAM5
MYBBP1A
CHEK2
CRYBA1
SIK1
E2F1
AKT1
DAPK2
MTOR
BRMS1
ITGA5
ITGB1
MCL1
CEACAM6
NOTCH1
NTRK2
PTRH2
PDK4
PIK3CA
ZNF304
PTK2
BCL2
SNAI2
SRC
STK11
MAP3K7
TFDP1
TLE1
TSC2
ANKRD13C
IKBKG
CAV1
BMF

Supplementary Table 2. The gene-specific primer pairs.

PDK4	
F	AGAGGTGGAGCATTTCTCGC
R	ATGTTGGCGAGTCTCACAGG
STK11	
F	CACTCAGGACTTCACGGTGC
R	CTCTGTGCCGTTCATACACAC
